# The effect of orthokeratology lens optical zone size on myopia control in adolescents

**DOI:** 10.3389/fmed.2026.1824676

**Published:** 2026-06-10

**Authors:** Lu Lai, Wen Long, Bingru Zheng, Ziqi Liang, Zijia He, Lu Chen, Dongmei Cui

**Affiliations:** Shenzhen Eye Hospital, Shenzhen Eye Medical Center, Southern Medical University, Shenzhen, China

**Keywords:** axial length elongation, back optic zone diameter, high defocus, myopia, Ortho-K lens

## Abstract

**Background:**

This study aims to investigate the impact of different optical zone sizes in orthokeratology (Ortho-K) lenses on myopia control in adolescents.

**Methods:**

A retrospective study was conducted on 84 subjects who had undergone Ortho-K lens treatment for over 12 months. Subjects were divided into two groups based on the optical zone size: 5-mm and 6-mm. The study compared changes in axial length, corneal curvature, corneal astigmatism, nasal defocus (ΔN), temporal defocus (ΔT), and the diameter of the peripheral plus power ring (PPRD). Additionally, corneal safety indicators, including corneal staining rate, corneal endothelial cell density, and visual acuity, were evaluated.

**Results:**

Data from the right eyes of 43 patients in the 5-mm group and 41 patients in the 6-mm group were analyzed. Baseline axial lengths were 24.77 ± 0.64 mm and 24.87 ± 0.75 mm, respectively. After 12 months of Ortho-K lens wear, the mean change in axial length was 0.25 ± 0.17 mm in the 5-mm group and 0.29 ± 0.16 mm in the 6-mm group, Repeated measures ANOVA revealed a significant time effect (*F* = 48.634, *P* < 0.001), a significant group effect (*F* = 4.041, *P* = 0.048), and a non-significant interaction effect between time and group (*F* = 2.361, *P* = 0.101). Multivariate linear regression analysis showed that age was negatively correlated with axial elongation (β = −0.557, 95% CI: −0.067 to −0.036, *P* < 0.001), while PPRD was positively correlated (β = 0.332, 95% CI: 0.055–0.163, *P* < 0.001). No significant differences were observed in safety indicators, including corneal staining rate, corneal endothelial cell density, and visual acuity.

**Conclusion:**

For adolescents with low-to-moderate astigmatism, 5-mm Back Optical Zone Diameter (BOZD) Ortho-K lenses demonstrated slightly superior efficacy in controlling myopia progression compared to 6- mm lenses, characterized by a smaller PPRD and higher defocus quantity. Measuring PPRD 1 month after lens wear may predict future axial length control.

## Introduction

It is projected that by 2050, nearly 5 billion people globally will suffer from myopia (1). Thus, early and effective management of low-to-moderate myopia progression is crucial. Orthokeratology (Ortho-K), a well-known non-surgical method, is widely used for myopia correction and has proven effective in curbing adolescent myopia progression (2). As a minimally invasive alternative, it has drawn significant attention in clinical practice and research due to its favorable outcomes and patient-friendly nature (2–9).

The mechanism of Ortho-K in slowing myopia progression is unclear, with peripheral defocus being the most accepted theory. It suggests that Ortho-K’s controlled corneal deformation causes a peripheral defocusing effect, potentially modulating visual inputs and altering ocular growth (10–12). Ortho-K lenses press the central cornea and suction the peripheral part, redistributing corneal epithelial cells and thinning the central cornea (13). This helps focus central light on the mid-peripheral retina and macula, while peripheral light focuses in front of the retina, creating myopic defocus, which may retard myopia progression (7, 14–16). Clinical research shows nighttime Ortho-K lens use significantly slows myopia (17–19).

Research on Ortho-K lenses dates back to the 1960s. Personalized design and new materials are at the forefront. A recent short-term study on a novel design found that Ortho-K lenses with a reduced back optic zone diameter (BOZD) enhanced peripheral defocus, increased higher-order aberrations (HOA), reduced treatment zones (TZ), and significantly limited axial elongation (20–22).

Although previous studies have thoroughly compared the control effects of CRT 5-mm BOZD and CRT 6-mm BOZD lenses on myopia progression, no further grouping and comparative analysis has been conducted between conventional and dual vector height Ortho-K lenses. It is well-documented that the treatment zone of Ortho-K lenses often becomes eccentric during treatment. Dual vector height design in Ortho-K lenses is one approach to maintain lens centration. Due to its unique design, CRT Ortho-K lenses with dual vector height are more commonly used than VST-design Ortho-K lenses. The impact of lens decentration on axial length changes in myopic children using Ortho-K lenses remains controversial. However, post-Ortho-K ghosting, which is associated with optical zone decentration, can compromise visual quality. Good daily visual acuity is crucial for patient compliance. Currently, there is limited detailed research analyzing the performance of dual vector height CRT lenses.

This study aims to deeply explore CRT-designed Ortho-K lenses with 5-mm and 6-mm BOZD dual vector heights, investigating differences in key myopia control indicators, especially axial length (AL) regulation, astigmatism rectification, and wearing safety. It is expected to provide clinicians with more precise and actionable guidance for lens fitting.

## Materials and methods

### Subjects

This retrospective study encompassed patients fitted with Paragon CRT (Paragon CRT 100 (paflufocon) Rigid Gas Permeable Contact Lenses for Corneal Refractive Therapy) orthokeratology lenses at the myopia prevention and control clinic of Shenzhen Eye Hospital between December 2021 to August 2024, 5-mm BOZD dual vector height and 6-mm BOZD dual vector height a period exceeding 12 months.

Inclusion criteria for the study were as follows:

1. Age range: 8–16 years inclusive; gender unrestricted;
2. The refractive power of any contact lens for one eye of the patient was within the applicable parameters of the study lenses (myopia within −4.00D inclusive and astigmatism total within 2.25D inclusive).”
3. Completed a 12-month follow-up visit;
4. The patient exhibited good lens wearing compliance, cooperated effectively with various eye examinations, and provided comprehensive initial fitting examination data;
5. During follow-up, records of axial length data were thoroughly documented, including at least three follow-up assessments;

The exclusion criteria for this study were as follows:

1. Subjects presenting with organic ocular pathologies, including but not limited to congenital amblyopia, strabismus, moderate to severe dry eye, cataract, and fundus diseases.
2. Systemic diseases affecting refractive development, such as connective tissue disorders, Down syndrome, Marfan syndrome.
3. Participants who utilized medications that have been scientifically validated to effectively impede myopia progression (e.g., low-concentration atropine eye drops, atropine gel) during the lens-wearing period. Alternatively, those who employed drugs potentially influencing corneal curvature (e.g., immunosuppressants, intraocular pressure-reducing agents) or altered lens parameters within 12 months of commencing lens wear.

This study was conducted in adherence to the principles of the Declaration of Helsinki, and received approval from the Ethics Committee of Shenzhen Eye Hospital (2024KYPJ047).

### Procedures

All patients underwent a comprehensive baseline ocular examination prior to fitting with Ortho-K lenses, including slit-lamp examination, uncorrected visual acuity measurement, best-corrected visual acuity (BCVA), axial length measurement, corneal endothelial cell examination, corneal topography examination.

Cycloplegic subjective refraction was performed before the initial fitting, using three drops of tropicamide-containing eye drops (0.5% tropicamide; SINQi, CHINA) with a 5-min interval between each drop to achieve cycloplegia. 30 min after the application of the third drop, three automated refractions were performed (NIDEK, Japan, model: ARK-1a), and the average value was calculated, followed by subjective refraction conducted by a professional optometrist.

Axial length (AL) measurements were conducted using the Optical Coherence Biometry 700 (IOL-Master 700; Carl Zeiss, Germany).

Corneal topography (Medmont E300U, Medmont International Pty Ltd., Australia) was assessed by the same dedicated professional technician to acquire precise corneal parameters. To analyze the corneal topography using Medmont software, begin by opening the selected map in the tangential map mode and identifying the plus power ring (PPR). Place a horizontal line at the center of the PPR, ensuring it intersects the widest part of the region. Utilize the cursor tool to move horizontally from the nasal to the temporal side, observing and recording the refractive power values in real-time. Identify and record the following key points: the lowest refractive power at the central cornea (central minimum point), the maximum refractive power in the nasal region (MaxN), and the maximum refractive power in the temporal region (MaxT). Calculate the nasal defocus (△N) as MaxN minus the central minimum value and the temporal defocus (△T) as MaxT minus the central minimum value. Measure the straight-line distance between MaxN and MaxT using the distance measurement tool, representing the diameter of plus power ring (PPRD) (23). All measurements are collected independently by two qualified professionals, and the final results are recorded as the mean of their measurements.

The corneal endothelial parameters (SW-7000, Suowei Electronic Technology Co., Ltd., China) were assessed by the same skilled professional technician to acquire precise corneal parameters.

The lens fitting was performed by the same experienced clinician, who selected the appropriate orthokeratology lenses based on the examination results of the myopic children and the manufacturer’s recommended guidelines. All participants were fitted were Paragon CRT 100 [paflufocon] Rigid Gas Permeable Contact Lenses. All lenses featured dual vector height design and differed exclusively in back-optical-zone diameter (BOZD): either 5 or 6 mm. Dual vector height refers to the design of contact lenses where the sagittal differences between the reverse and landing zones are created along the two principal meridians of the lens, aiming to improve lens positioning and wearing stability. Based on the prescribed BOZD, participants were retrospectively categorized into two mutually exclusive groups—the 5-mm BOZD group and the 6-mm BOZD group—enabling direct comparison of treatment outcomes associated with each BOZD configuration.

Following the initiation of orthokeratology lens wear, all patients were advised to wear the lenses continuously for a minimum of 8 h each night. Following orthokeratology lens wear, slit-lamp ocular examination, BCVA measurement, and corneal topography examination were conducted at each follow-up visit, including on 1 day, 1 week, 1 month, 3 month, and every 3 months thereafter. Axial length measurement was conducted at 6 and 12 months post-fitting. Corneal endothelial cell examination is conducted once before commencement and again at 12 months post-commencement of lens wear.

### Statistical analysis

To minimize potential bias arising from binocular interaction, this study exclusively evaluated the right eye of the subjects. This approach ensures data independence and enhances the accuracy and reproducibility of the results.

The normality of continuous variables was assessed using the Shapiro-Wilk test. Continuous variables are presented as mean ± standard deviation when appropriate).

Repeated measures ANOVA was used to analyze AL, FK, DK, and VA at baseline and each follow-up visit. To evaluate factors potentially influencing axial length growth, a univariate analysis was first conducted, including age, gender, spherical equivalent (SE), corneal curvature, PPRD, ΔN, ΔT, BOZD, and Corneal Elevation Difference. Variables with p < 0.05 in the univariate analysis were then included in a multivariate linear regression model.

All statistical analyses were conducted using SPSS software (version 29.0; SPSS Inc., NY, United States). The significance level was set at a two-tailed *p* < 0.05.

## Results

A total of 84 participants were included in this retrospective study: 43 in the 5-mm BOZD dual vector height group and 41 in the 6-mm BOZD dual vector height group.

The average age was 10.12 ± 1.85 years for the 5-mm BOZD dual vector height group, 10.39 ± 1.95 years for the 6-mm BOZD dual vector height group. The mean SE was −2.89 ± 1.21 D for the 5-mm BOZD dual vector height group, −2.77 ± 0.95 D for the 6-mm BOZD dual vector height group.

The 5-mm BOZD dual vector height group did not show statistically significant differences in age, gender, baseline axial length, FK, and SK compared to the 6-mm BOZD dual vector height group (Table 1).

**TABLE 1:** Biometric measurements of study subjects.

Variables	Group 1	Group 2	
	5-mm BOZD	6-mm BOZD	*P*-value*
n	43	41	
Age (years)	10.12 ± 1.85	10.39 ± 1.95	0.511
Gender (male/female)	16/27	21/20	0.205
SE (D)	−2.89 ± 1.21	−2.77 ± 0.95	0.612
Baseline AL (mm)	24.77 ± 0.64	24.87 ± 0.75	0.506
Flat K (D)	43.08 ± 1.09	43.07 ± 1.10	0.964
Steep K (D)	44.45 ± 1.22	44.47 ± 1.18	0.958
Corneal elevation difference	29.71 ± 10.80	33.80 ± 8.66	0.06

SE, Spherical equivalent refractive error; AL, Axial length; Flat K, Flat Keratometry; Steep K, Steep Keratometry, Corneal Elevation Difference Refers to the corneal elevation difference between the cornea and the lens within an 8 mm chord length.mm, millimeter; D, diopter. * Chi-Square test for the gender, and independent sample *t* −test for the others.

### Axial length

At 6 months, AL increases were 0.12 ± 0.16 mm in the 5-mm BOZD dual-vector height group and 0.16 ± 0.08 mm in the 6-mm BOZD dual-vector height groups. At 12 months, mean AL increases were 0.25 ± 0.17 mm and 0.29 ± 0.16 mm, respectively. Repeated measures ANOVA was used to evaluate changes in axial length at baseline, 6 months, and 12 months after lens wear. The main effect of time on axial length change was significant (*F* = 48.634, *P* < 0.001), and the main effect of group was also significant (*F* = 4.041, *P* = 0.048). However, the interaction effect between time and group was not significant (*F* = 2.361, *P* = 0.101) (Table 2).

**TABLE 2:** Changes in AL from baseline to 6 and 12 months after Ortho-K lens wear.

Group	Baseline (mm)	6 months (mm)	12 months (mm)	AL change 6 m (mm)	AL change 12 m (mm)
5-mm BOZD	24.77 ± 0.64	24.91 ± 0.63	25.02 ± 0.63	0.12 ± 0.16	0.25 ± 0.17
6-mm BOZD	24.87 ± 0.75	25.00 ± 0.73	25.16 ± 0.73	0.17 ± 0.10	0.29 ± 0.17

AL, Axial length; mm, millimeter. F_*time*_ = 48.634, *p* = < 0.001, F_*group*_ = 4.041, *p* = 0.048, F_*intra*_ = 2.361, *p* = 0.101.

### The diameter of plus power ring

After 1 month of orthokeratology lens wear, the Plus power ring(PPR)was fully established in both groups. No statistically significant differences in PPRD were observed between the 1-, 3-, 6-, and 12-month follow-up points within each group. Repeated measures ANOVA demonstrated that the PPRD at all follow-up points was significantly smaller in the 5-mm BOZD group compared to the 6-mm group. Both groups exhibited asymmetric PPR values, with significantly higher mean nasally directed PPR values than temporally directed PPR values. Repeated measures ANOVA was conducted to compare the ΔN and ΔT at each follow-up point. The 5-mm group showed slightly higher nasal and temporal defocus values compared to the 6-mm group, but these differences were not statistically significant (Tables 3–5).

**TABLE 3:** The size of the nasally defocus amount after wearing Ortho-K lenses for 1, 3, 6, and 12 months.

Group	1 months (D)	3 months (D)	6 months (D)	12 months (D)
5**-**mm BOZD	12.00 ± 4.08	12.15 ± 4.53	11.76 ± 4.71	10.54 ± 3.69
6**-**mm BOZD	10.94 ± 3.57	10.82 ± 3.69	10.42 ± 3.82	10.70 ± 3.32
*p*	0.101	0.634	0.365	0.973

Nasally defocus amount (ΔN),F_*time*_ = 4.238, *p* = 0.006, F_*group*_ = 0.929, *p* = 0.338, F_*intra*_ = 1.164, *p* = 0.324.

**TABLE 4:** The size of the temporal defocus amount after wearing Ortho-K lenses for 1, 3, 6, and 12 months.

Group	1 months (D)	3 months (D)	6 months (D)	12 months (D)
5**-**mm BOZD	9.58 ± 2.98	9.59 ± 3.04	9.34 ± 3.97	8.22 ± 2.80
6**-**mm BOZD	8.47 ± 2.81	8.99 ± 3.30	8.33 ± 3.24	8.42 ± 2.56
*p*	0.234	0.286	0.295	0.863

Temporal defocus amount (ΔT), F_*time*_ = 2.497, *p* = 0.06, F_*group*_ = 1.147, *p* = 0.287, F_*intra*_ = 0.648, *p* = 0.585.

**TABLE 5:** The size of the PPRD after wearing Ortho-K lenses for 1, 3, 6, and 12 months.

Group	1 months (D)	3 months (D)	6 months (D)	12 months (D)
5**-**mm BOZD	4.71 ± 0.51	4.69 ± 0.49	4.80 ± 0.54	4.69 ± 0.49
6**-**mm BOZD	5.16 ± 0.54	5.14 ± 0.52	5.19 ± 0.53	5.13 ± 0.58
*p*	< 0.001	<0.001	0.003	< 0.001

Diameter of plus power ring (PPRD), F_*time*_ = 1.391, *p* = 0.246, F_*group*_ = 16.482, *p* = < 0.001, F_*intra*_ = 0.264, *p* = 0.851.

### Regression analysis of factors influencing axial elongation

Univariate linear regression analysis was performed to assess the association between age, sex, baseline spherical equivalent (SE), baseline flat keratometry (FK), baseline steep keratometry (DK), baseline height difference, BOZD, post-wear 1-month PPRD, post-wear 1-month change in ΔT, post-wear 1-month change in ΔN, and axial length growth after 12 months of lens wear. The results showed that age was negatively associated with axial length growth (β = −0.59, 95% confidence interval [CI]: −0.071 to −0.038, *P* < 0.001), and PPRD was positively associated with axial length growth (β = 0.387, 95% CI: 0.061–0.194, *P* < 0.001). No significant associations were found for the other variables (all *P* > 0.05). In the multivariate linear regression analysis, age remained negatively associated with axial length growth (β = −0.557, 95% CI: −0.067 to −0.036, *P* < 0.001), and PPRD remained positively associated with axial length growth (β = 0.332, 95% CI: 0.055–0.163, *P* < 0.001) (Table 6).

**TABLE 6:** Linear regression analysis of factors influencing axial length growth after wearing orthokeratology lenses.

Parameters	Univariate analysis			Multivariate analysis		
	β	95%CI	*P*	β	*95%CI*	*P*
Age (years)	−0.59	−0.071 to −0.038	< 0.001	−0.557	−0.067 to −0.036	< 0.001
Gender	−0.003	−0.078 to 0.076	0.977			
SE (D)	0.101	−0.02 to 0.054	0.36			
Flat K (D)	0.018	−0.032 to −0.289	0.773			
Steep K (D)	−0.059	−0.041 to 0.023	0.592			
Corneal elevation difference	−0.052	−0.005 to 0.003	0.637			
BOZD	0.13	−0.031 to 0.121	0.238			
PPRD	0.387	0.061-0.194	< 0.001	0.332	0.055-0.163	< 0.001
ΔN	0.106	−0.007 to 0.02	0.336			
ΔT	0.163	−0.003 to 0.018	0.138			

SE, Spherical equivalent refractive error; Flat K, Flat Keratometry; Steep K, Steep Keratometry, Corneal Elevation Difference Refers to the corneal elevation difference between the cornea and the lens within an 8 mm chord length. BOZD, Back Optic Zone Diameter; PPRD, Diameter of plus power ring; ΔT, Temporal defocus amount; ΔN, nasally defocus amount; mm, millimeter; D, diopter.

### Corneal curvature

Throughout a 12-month research period, the orthokeratology lens group exhibited substantial corneal reshaping.

After 12 months, the flat K readings in the 5-mm BOZD dual vector height group decreased from 43.08 ± 1.09 D to 41.46 ± 1.29 D; in the 6-mm BOZD dual vector height group, from 43.07 ± 1.10 D to 40.79 ± 1.21 D.

Regarding steep K values, the 5-mm BOZD dual vector height group decreased from 44.45 ± 1.22 D to 42.81 ± 1.34 D; the 6-mm BOZD dual vector height group from 44.47 ± 1.18 D to 42.01 ± 1.16 D.

**FIGURE 1:**
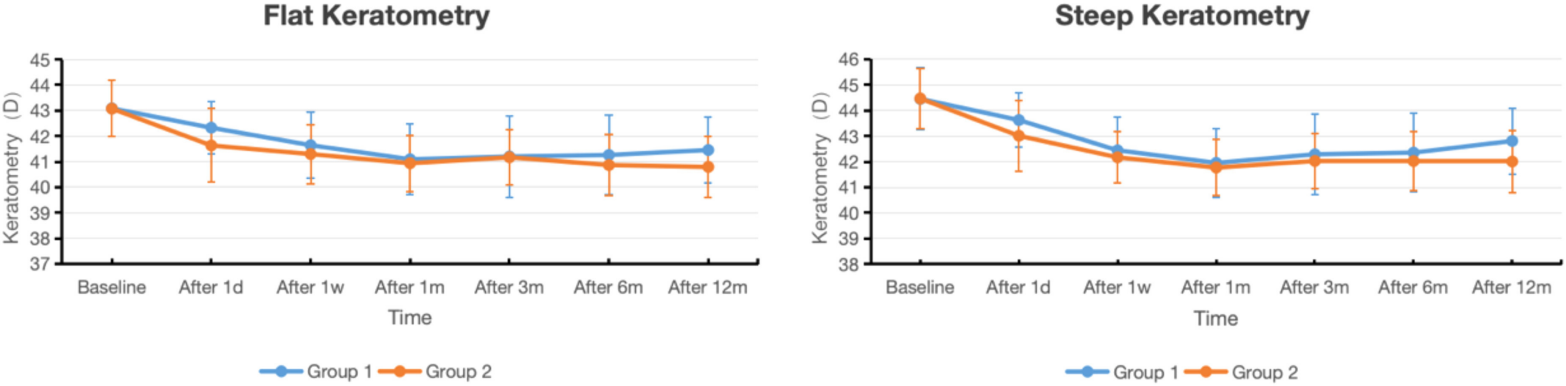
Corneal keratometry changes between the two groups. Group 1: 5-mm BOZD; Group 2: 6-mm BOZD.

The corneal curvature measurements at different observation points are detailed in Figure 1.

*Post-hoc* analysis indicated that the cornea had significantly flattened at all follow-up points relative to baseline (*p* < 0.05). Repeated measures ANOVA revealed a significant main effect of time on changes in flat keratometry (FK) (*F* = 87.716, *P* < 0.001), a significant main effect of group (*F* = 5.499, *P* = 0.021), and a significant interaction effect between time and group (*F* = 56.263, *P* < 0.001). Similarly, for steep keratometry (DK), there was a significant main effect of time (*F* = 96.399, *P* < 0.001), a significant main effect of group (*F* = 6.100, *P* = 0.016), and a significant interaction effect between time and group (*F* = 53.460, *P* < 0.001).

After the first week and 12 months post-wearing, it was observed that the alterations in both flat and steep corneal curvatures were less pronounced in the 5-mm BOZD dual vector height group compared to the 6-mm BOZD dual vector height group, with statistically significant differences noted (*p* < 0.05).

### Corneal astigmatism

This study investigated the impact of orthokeratology lens wear on corneal astigmatism. While both types of lenses resulted in varying degrees of corneal astigmatism reduction, no significant differences were observed between the groups at any follow-up points. Repeated measures ANOVA revealed a significant main effect of time on corneal astigmatism (*F* = 30.784, *P* < 0.001), no significant main effect of group (*F* = 0.157, *P* = 0.693), and a significant interaction effect between time and group (*F* = 2.242, *P* = 0.048).

**FIGURE 2:**
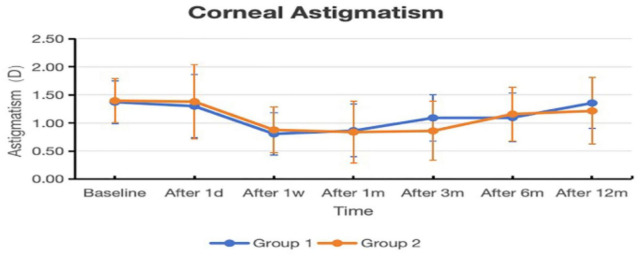
corneal astigmatism changes between the two groups. Group 1: 5-mm BOZD; Group 2: 6-mm BOZD.

Corneal astigmatism measurements at various follow-up points are shown in Figure 2.

### Visual acuity (VA)

One week after orthokeratology lens wear initiation, both groups achieved excellent uncorrected visual acuity (UCVA), with no statistically significant difference between groups (p > 0.05). Changes in visual acuity (VA) at each observation point, recorded in logMAR, are detailed in Table 7.

**TABLE 7:** Visual acuity (logMAR) after orthokeratology at 1, 6, and 12 months visits.

Time	5-mm BOZD	6-mm BOZD	*P*-value
1 day	0.18 ± 0.17	0.17 ± 0.17	0.012
1 week	0.03 ± 0.07	0.01 ± 0.03	0.032
1 month	0.02 ± 0.06	0.01 ± 0.05	0.189
3 months	0.02 ± 0.06	0.02 ± 0.10	0.959
6 months	0.04 ± 0.16	0.01 ± 0.04	0.313
12 months	0.02 ± 0.06	0.04 ± 0.10	0.105

F_*time*_ = 14.479, *p* = < 0.001, F_*group*_ = 0.461, *p* = 0.499, F_*intra*_ = 0.662, *p* = 0.653.

### Endothelium and corneal staining

No statistically significant difference was observed in baseline corneal endothelial parameters between the two groups and after 12 months of orthokeratology lens wear (*p* ≥ 0.05) (Table 8). Most corneal staining was grade 0 or 1 and resolved spontaneously without intervention. Corneal Epithelial Punctate Staining Grading According to the CCLRU Standards (24).

**TABLE 8:** Changes in corneal endothelial parameters from baseline to 12 months after Ortho-K lens wear.

TIME	5-mm BOZD			6-mm BOZD		
	CD(/mm^2^)	CV(%)	6A(%)	CD(/mm^2^)	CV(%)	6A(%)
Baseline	3246.26 ± 240.45	36.47 ± 4.84	59.47 ± 7.1	3194.98 ± 230.82	35.8 ± 6.11	62.2 ± 7.21
12 months	3227.49 ± 247.67	36.54 ± 5.33	59.44 ± 6.39	3189.44 ± 234.32	35.69 ± 4.27	60.14 ± 6.6

CD, endothelial cell density; CV, coefficient of variation; 6A hexagonal cell ratio.

Among these patients, in the 5-mm BOZD dual vector height group, 23 eyes experienced one or more episodes of corneal staining (9 eyes had one episode; 7 eyes had two episodes; 7 eyes had three or more episodes). In the 6-mm BOZD dual vector height group, 23 eyes experienced one or more episodes of corneal staining (15 eyes had one episode; 5 eyes had two episodes; 3 eyes had three or more episodes).

None of the patients discontinued lens wear due to corneal staining.

## Discussion

Previous prospective studies have consistently shown that orthokeratology (Ortho-K) lenses with a 5-mm back optic zone diameter (BOZD) are more effective in myopia control than those with a 6 mm BOZD. Our retrospective analysis confirmed these findings. The axial elongation in the 5-mm BOZD dual vector height group was 0.04 mm less than that in the 6-mm BOZD dual vector height group.

Similar results were reported by Lin et al. Their 5-mm BOZD (DRL) lens group had a 0.15-mm reduction in annual axial elongation (53.6%) compared to the 6.2-mm BOZD (Euclid) lens group (25). Guo et al.’s RCT found that 5-mm BOZD Ortho-K lenses led to a 0.13-mm reduction in axial elongation (76.5%) relative to 6-mm ones, significant only in the first 6 months (22). Pauné et al. noted that smaller BOZD Ortho-K lenses could limit myopic progression by about 0.06 mm per year. A smaller BOZD may induce higher higher-order aberrations, potentially slowing axial length growth (26). Multiple studies suggest a smaller treatment zone may result in more effective myopia control (27–30).

However, our retrospective analysis using CRT-designed Ortho-K lenses showed no significant differences in axial length changes between the two patient groups at 6 and 12 months. The axial length changes in our groups were greater than in Guo et al.’s study, possibly due to differences in orthokeratology lens compliance between clinical patients and prospective study participants. A retrospective study of 238 subjects aged 7–25 years showed poor adherence to Ortho-K lens wear, with a compliance rate of 19.7% (31). The COVID-19 containment measures during the study period likely contributed to reduced compliance with orthokeratology lens wear and prolonged digital device use (e.g., online learning and recreational activities), compounded by insufficient outdoor light exposure. These environmental factors synergistically accelerated myopic progression, potentially diminishing the anticipated efficacy of orthokeratology in controlling myopia. Moreover, based on the criteria established by Wang et al., wherein subjects with less than 0.3 mm of axial elongation after 1 year of orthokeratology were defined as effectively controlling myopia progression, both groups in this study were considered to have achieved effective myopia control (32).

Furthermore, the efficacy of orthokeratology lenses varies significantly among individuals. Nominal differences in optical zone diameter may be offset by individual corneal curvature and lens fitting characteristics, which influence the effective treatment zone. The actual optical zone on the cornea does not always match the BOZD, thereby diminishing the theoretical control advantages.

The impact of PPRD and defocus on axial length is well-documented. Previous studies have shown that the diameter of the corneal defocus ring and the amount of defocus within the pupil can influence the rate of axial elongation in myopic patients. Smaller defocus ring diameters and less defocus within the pupil are associated with slower axial elongation.

In this study, 1 month after lens wear, the mean defocus ring diameter in the 5-mm group (4.71 ± 0.51 mm) was significantly smaller than that in the 6-mm group (5.16 ± 0.54 mm). After 1 month, the nasal side showed significantly higher defocus values than the temporal side. For the 5-mm group, the nasal defocus value was 12.00 ± 4.08 diopters (D), and the temporal defocus value was 9.58 ± 2.98 D. For the 6-mm group, the nasal defocus value was 10.94 ± 3.57 D, and the temporal defocus value was 8.47 ± 2.81 D. For the 6-mm group, the temporal defocus value was 15.87 ± 10.94 D, and the nasal defocus value was 8.47 ± 2.81 D. Repeated measures ANOVA indicated that the defocus values in the nasal and temporal sides of the 5-mm group were slightly higher at each follow-up point compared to the 6-mm group, but the differences were not statistically significant. Although there were statistically significant differences in PPRD between the two groups, no significant differences were observed in the defocus values, which are a key indicator of myopia control. This suggests that the peripheral myopic defocus optical stimuli provided by both groups were essentially equivalent and did not result in a significant difference in the effective dose for inhibiting axial elongation. Queiros further measured the peripheral refractive state along the horizontal axis after orthokeratology treatment and found relative hyperopic defocus (flattening) in the ± 20° regions relative to the baseline, no changes at 25°, and myopic defocus beyond 25° (15). In a study involving young monkeys, it was found that peripheral defocus beyond 20° from the fovea had no effect on axial elongation control, suggesting that myopic defocus needs to be closer to the central fovea to influence myopia control (33). Reducing the BOZD resulted in a larger PPRD but did not significantly increase the defocus quantity or make it more prominent in the effective zone. This may be an important optical mechanism explaining the lack of statistically significant differences in axial length elongation between the two groups, which requires further investigation to confirm.

Our study confirms that there were no significant differences in PPRD between the 1-month and 3-, 6-, and 12-month post-wear data points within each group. Recent research suggests that PPRD is the optimal predictor of monthly axial length growth, with an increase in axial length growth rate as PPRD increases when PPRD ≥ 4.1244 mm (23). However, the PPRD values in that study were measured 12 months after orthokeratology lens wear. Our findings suggest that the predictive period for the effect of orthokeratology lenses on axial elongation could be advanced to 1 month post-wear. This, however, requires larger sample sizes and longer follow-up periods for validation.

Previous research has demonstrated that orthokeratology lenses correct vision predominantly by modifying the FK and SK corneal curvatures. Wearing these lenses usually flattens the central corneal curvature. This is primarily because the pressure distribution of the lenses on the cornea gradually declines, resulting in central corneal flattening.

In our 12-month study, at the end, the FK in the 5-mm BOZD dual-vector height group decreased by −1.63 ± 0.92 D, and in the 6-mm group, it decreased by −2.31 ± 0.78 D. The SK in the 5-mm group decreased by −1.64 ± 0.95 D, and in the 6-mm group, it decreased by −2.48 ± 0.96 D.

The study revealed that compared with the 6-mm BOZD dual-vector height orthokeratology lenses, the 5-mm ones caused less corneal curvature change but were more effective in myopia control. This finding implies that appropriately reducing the optical zone can more precisely and significantly impact myopia control.

There were no significant differences in visual acuity between the groups in this retrospective study. Good daily visual acuity is vital for patients’ compliance. Dual vector height designed Ortho-K lenses are ideal for correcting moderate-to-high myopic astigmatism. Their optical zone is spherical, while the reverse and/or fitting curves have a special dual vector height design, adapting to the peripheral cornea and improving stable fitting for high-astigmatism patients (34). This design ensures stable corneal positioning, reducing visual fluctuations and discomfort. It also evenly distributes pressure on the cornea, enhancing comfort and lowering the risk of corneal hypoxia.

Corneal endothelial cell assessment constitutes a critical component of long-term safety monitoring in individuals undergoing prolonged orthokeratology lens wear. In our 12-month study on two groups of myopic patients using orthokeratology lenses, we found no significant differences in endothelial cell density (CD), coefficient of variation (CV), and hexagonal cell ratio (6A) compared to baseline (*p* > 0.05), nor between the two groups (*p* > 0.05). These results confirm that long-term use of orthokeratology lenses does not significantly affect endothelial cell density, morphology, and average characteristics, suggesting their safety in myopia control. Although some complications like corneal epithelial damage may occur, in our study, all corneal staining cases were grade 0 or 1, not affecting lens wear, and no serious adverse events were reported for the two lens designs.

### Limitations

Retrospective design and follow-up: This study is retrospective, and the frequency and quality of patient follow-ups could not be controlled. Subjects were excluded for protocol-defined reasons—namely, failure to attend scheduled follow-up visits or incomplete data collection—resulting in a final small sample size of 84. Although all eligible participants meeting the prespecified inclusion and exclusion criteria were included, the retrospective study design and limited sample size may have introduced selection bias, residual confounding, and reduced statistical power—increasing the risk of type II error. However, the absence of pre-fitting longitudinal axial length growth data—specifically, the individual baseline growth rate prior to orthokeratology lens initiation—limits the precision of axial length control evaluation.

## Conclusion

A comprehensive 12-month retrospective analysis of clinical data indicates that 5-mm BOZD orthokeratology lenses have a slight advantage in slowing myopia progression in adolescents. The 5-mm BOZD group showed a smaller PPR and a trend toward better axial length control compared to the 6-mm BOZD group. Future studies should increase the sample size to validate these findings. Measuring PPRD 1 month after lens wear may predict future axial length control. No significant differences were observed between the groups in key safety parameters (p > 0.05). This study may guide clinicians in formulating myopia prevention and control plans for children with mild to moderate astigmatism and concurrent myopia.

## Data Availability

The raw data supporting the conclusions of this article will be made available by the authors, without undue reservation.
